# Supplementary figures and images for: Regional fresh snowfall microbiology and chemistry are driven by geography in storm-tracked events, Colorado, USA
Source: PeerJ. 2018 Nov 21;6:e5961. doi: 10.7717/peerj.5961 (PMC6252068; doi:10.7717/peerj.5961)

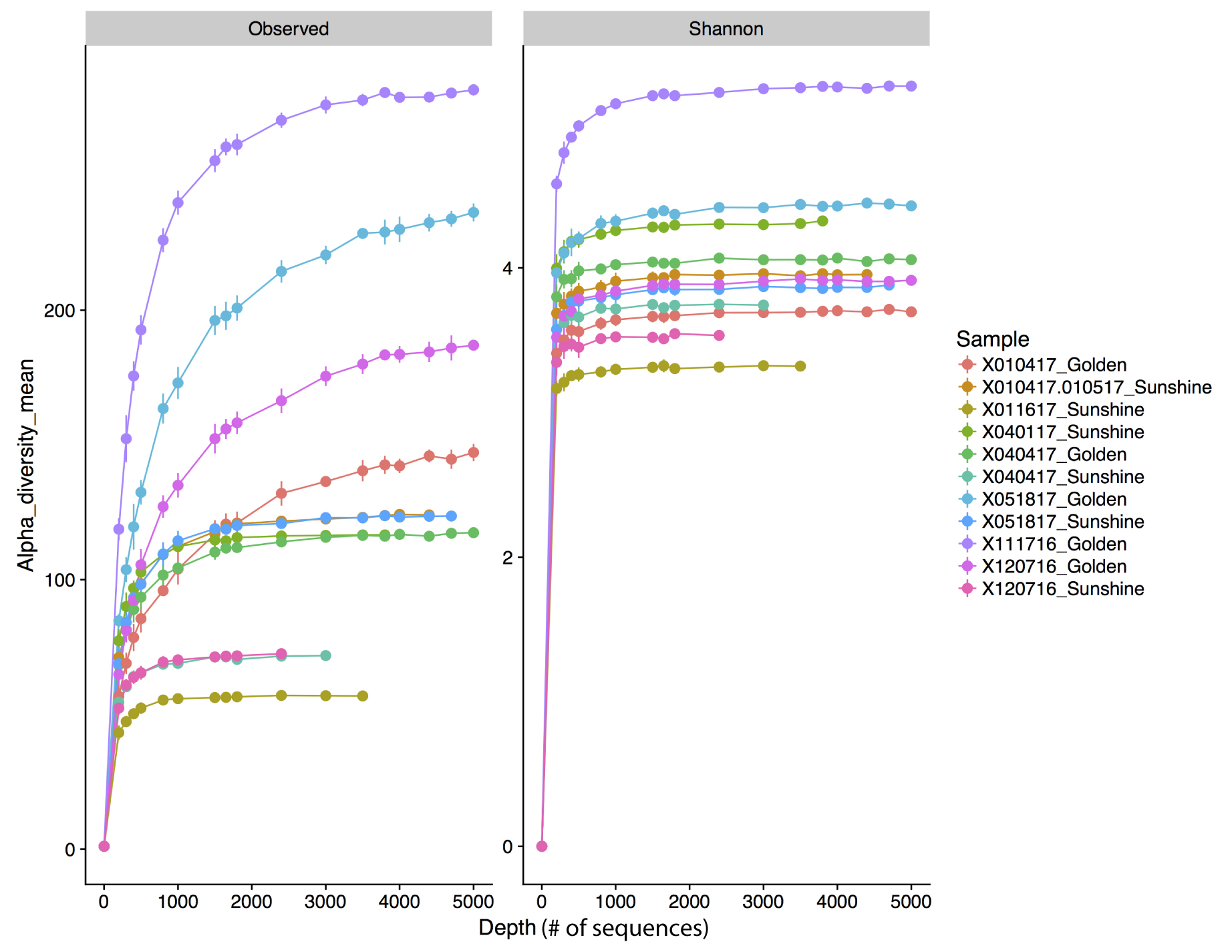

Supplement: Supplemental Information 1 — Rarefaction curves generated by rarefying bacterial/archaeal sequences at different levels with random OTU sub-sampling. Each curve and color represents a different sample. A blanket rarefaction value of 3,391 sequences was chosen for the bacterial and archaeal sequence dataset. [file peerj-06-5961-s001.jpg]

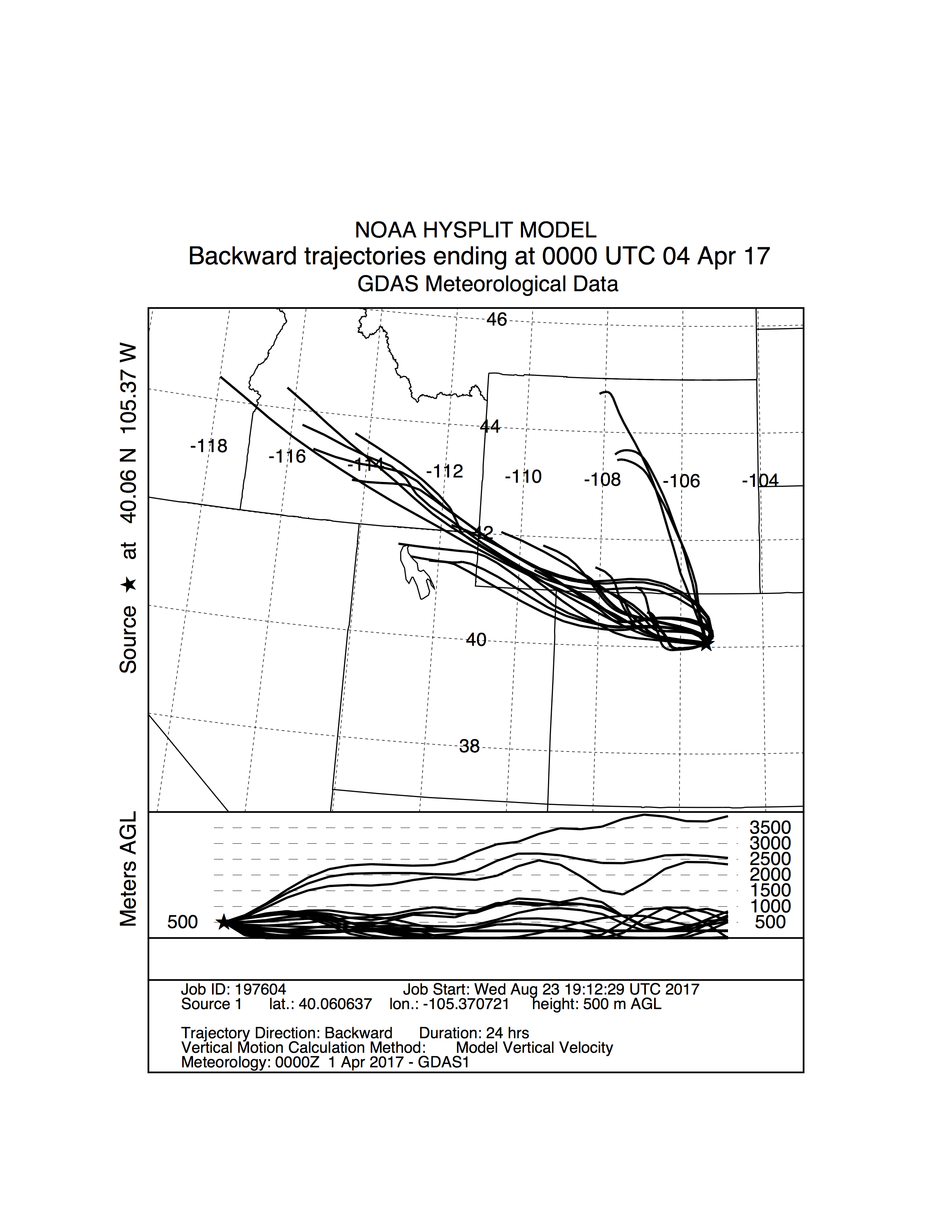

Supplement: Supplemental Information 2 — NOAA HYSPLIT storm trajectory model generated for the sample collection in Sunshine on 04/04/17. Streamlines are a medley of trajectory models distributed about the latitude and longitude of the sampling site. The backward trajectories encompass 24 hours of storm movement prior to arrival at the sampling location at an altitude of 500 m AGL (above ground level). This storm was classified as having a NW origin. See Fig. S3 for the Golden counterpart to this storm. [file peerj-06-5961-s002.jpg]

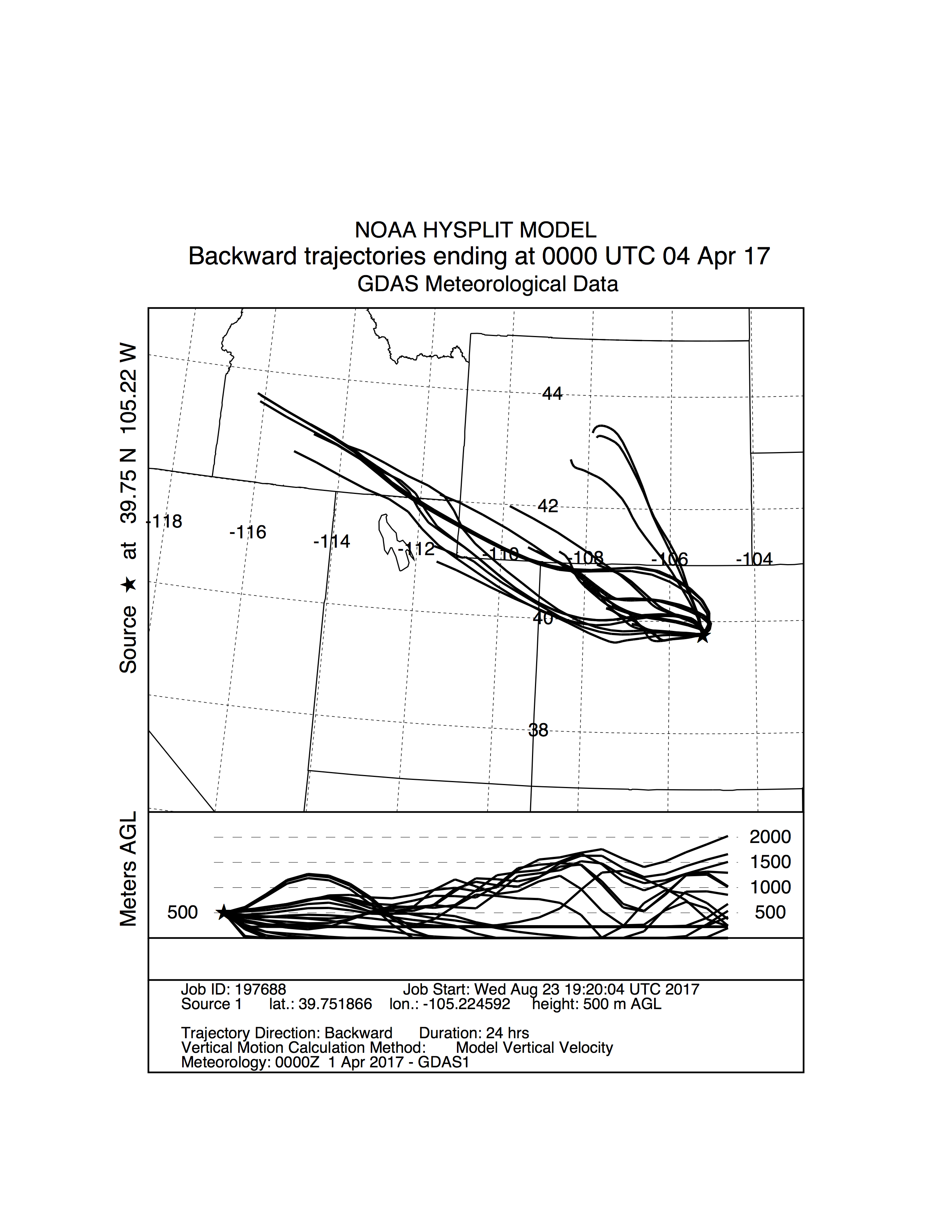

Supplement: Supplemental Information 3 — NOAA HYSPLIT storm trajectory model generated for the sample collection in Golden on 04/04/17. Streamlines are a medley of trajectory models distributed about the latitude and longitude of the sampling site. The backward trajectories encompass 24 hours of storm movement prior to arrival at the sampling location at an altitude of 500 m AGL (above ground level). This storm was classified as having a NW origin. See Fig. S2 for the Sunshine counterpart to this storm. [file peerj-06-5961-s003.jpg]

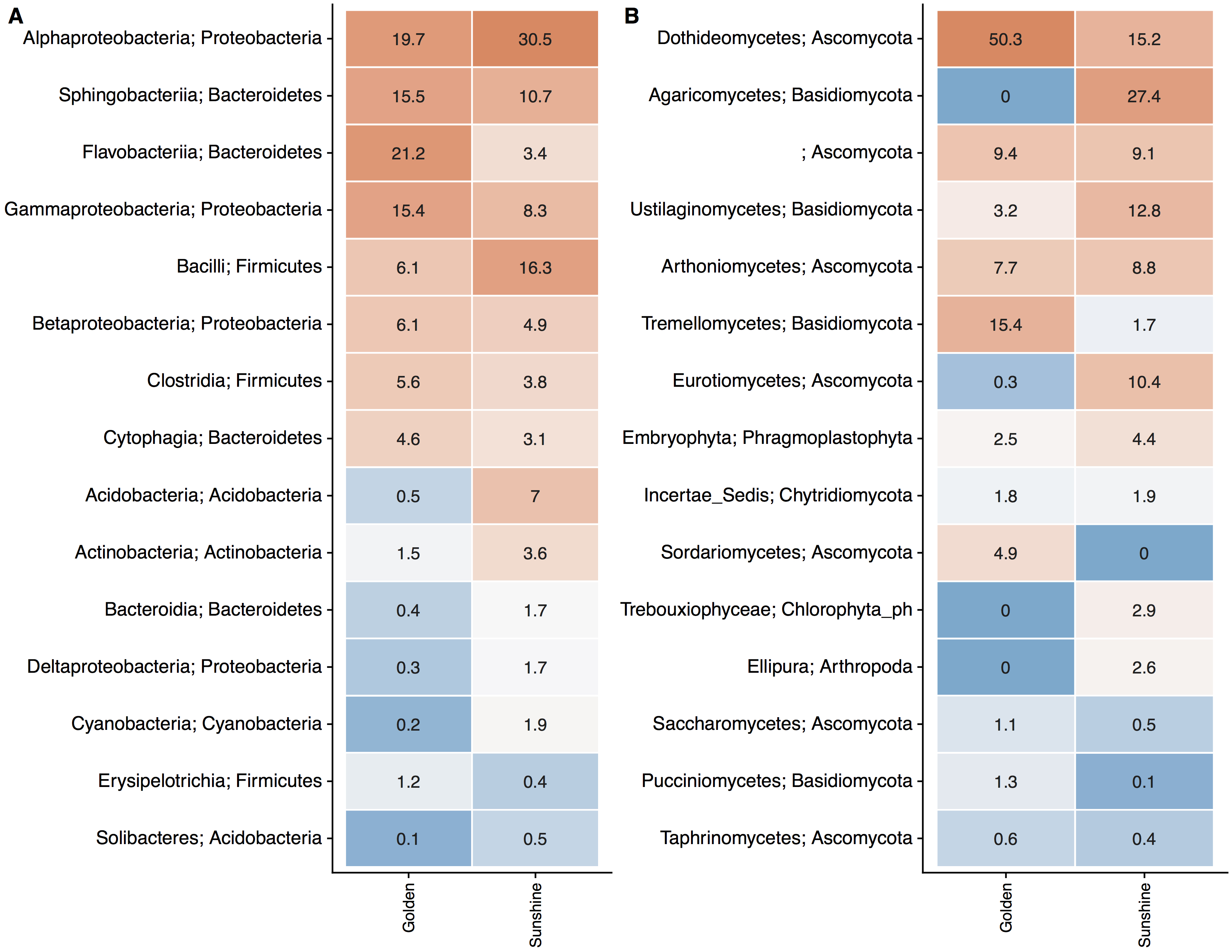

Supplement: Supplemental Information 4 — Relative abundances (more red = higher relative abundance) of bacterial/archaeal (A) and eukaryotic (B) sequences recovered from snow samples pooled by Sampling Location. Class-level assignments are listed first on the y-axis for each taxa, followed by a semi-colon and its associated phylum. Taxonomy was assigned via the SILVA database by DADA2. These data have been rarefied. [file peerj-06-5961-s004.jpg]

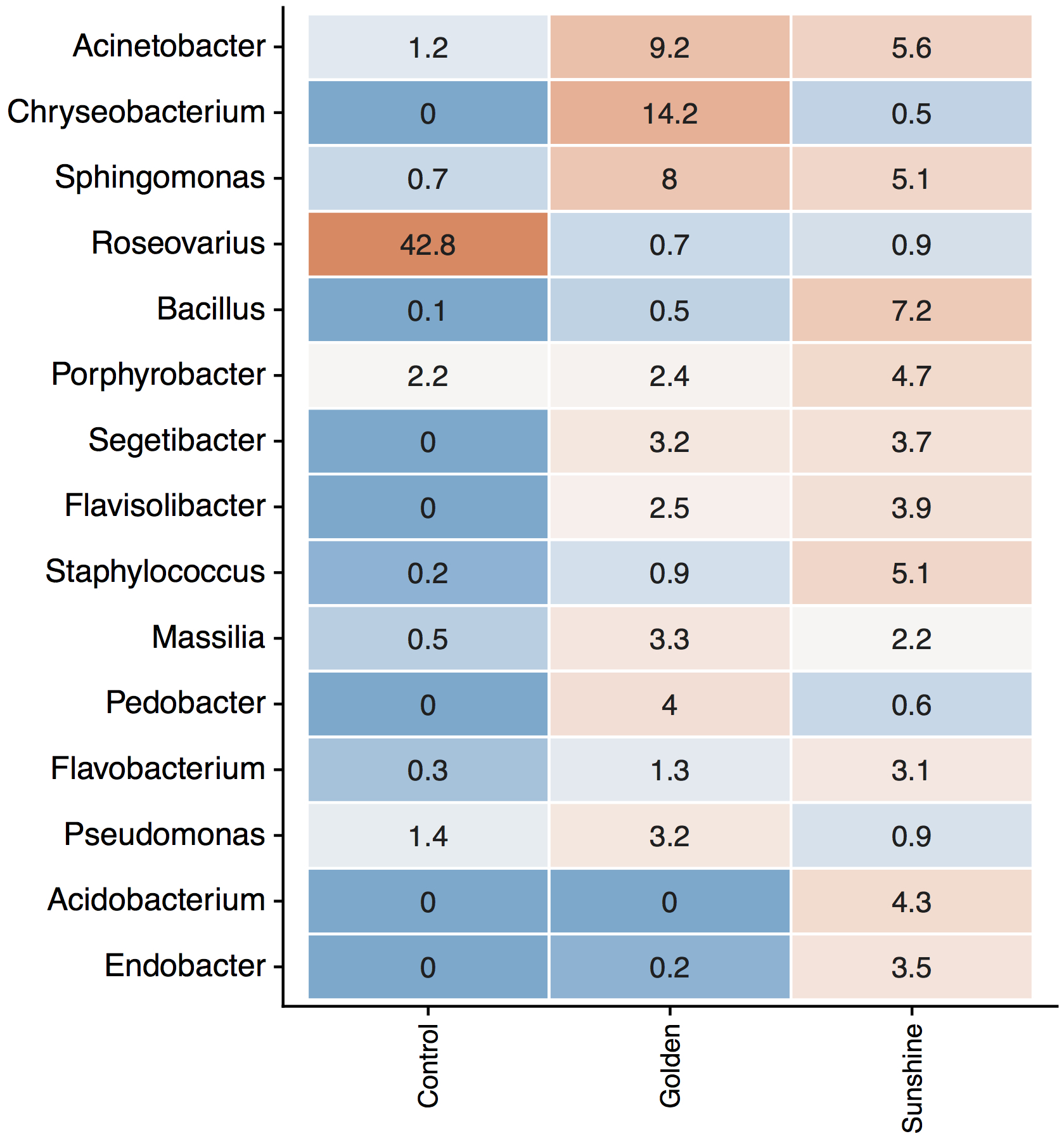

Supplement: Supplemental Information 5 — Demonstration that the most influential genera listed in Table 1 are not contamination. Numbers are relative abundances; more red means higher abundance. These data have been rarefied the same as all other analyzed samples. [file peerj-06-5961-s005.jpg]

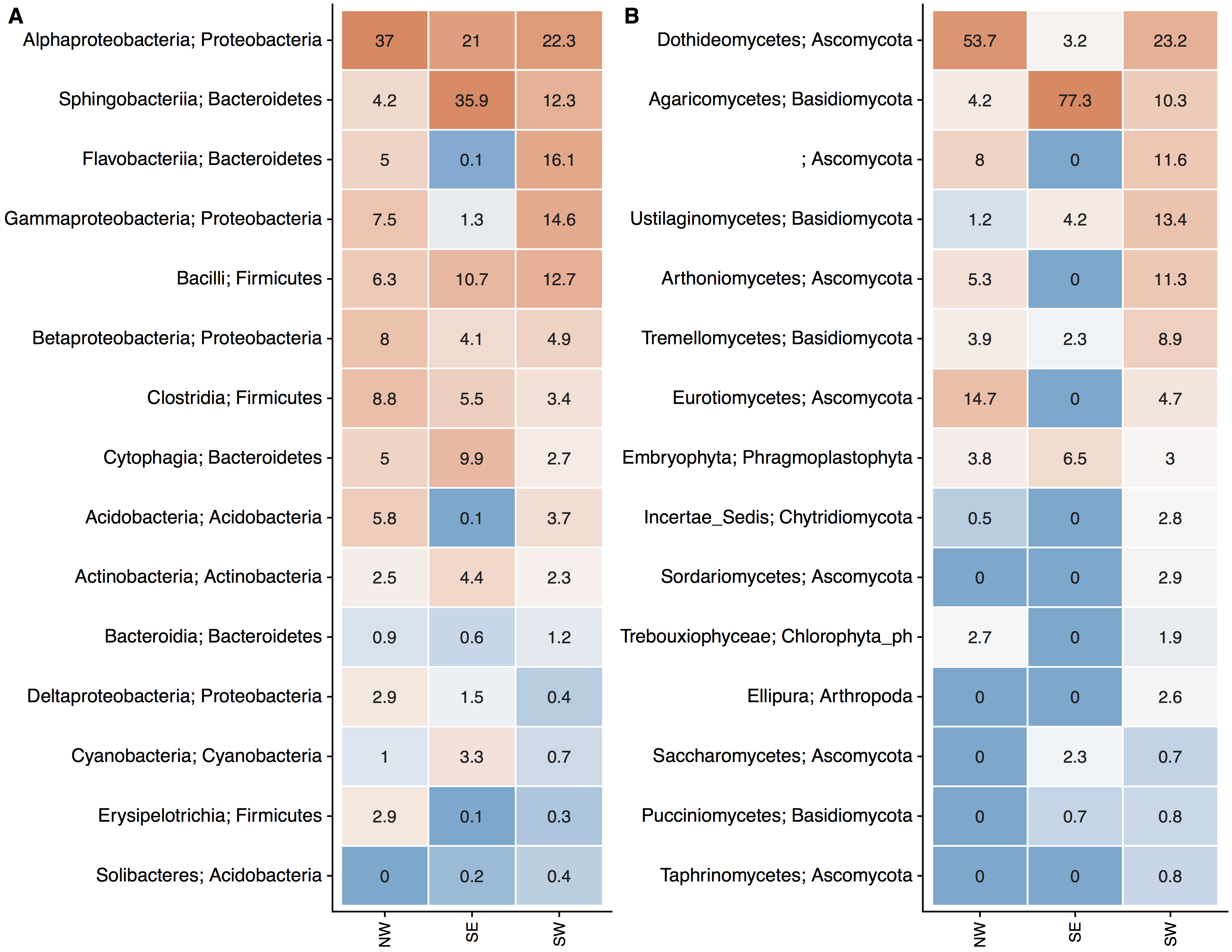

Supplement: Supplemental Information 6 — Relative abundances (more red = higher relative abundance) of bacterial/archaeal (A) and eukaryotic (B) sequences recovered from snow samples pooled by Storm Origin. Class-level assignments are listed first on the y-axis for each taxa, followed by a semi-colon and its associated phylum. Taxonomy was assigned via the SILVA database by DADA2. These data have been rarefied. [file peerj-06-5961-s006.jpg]

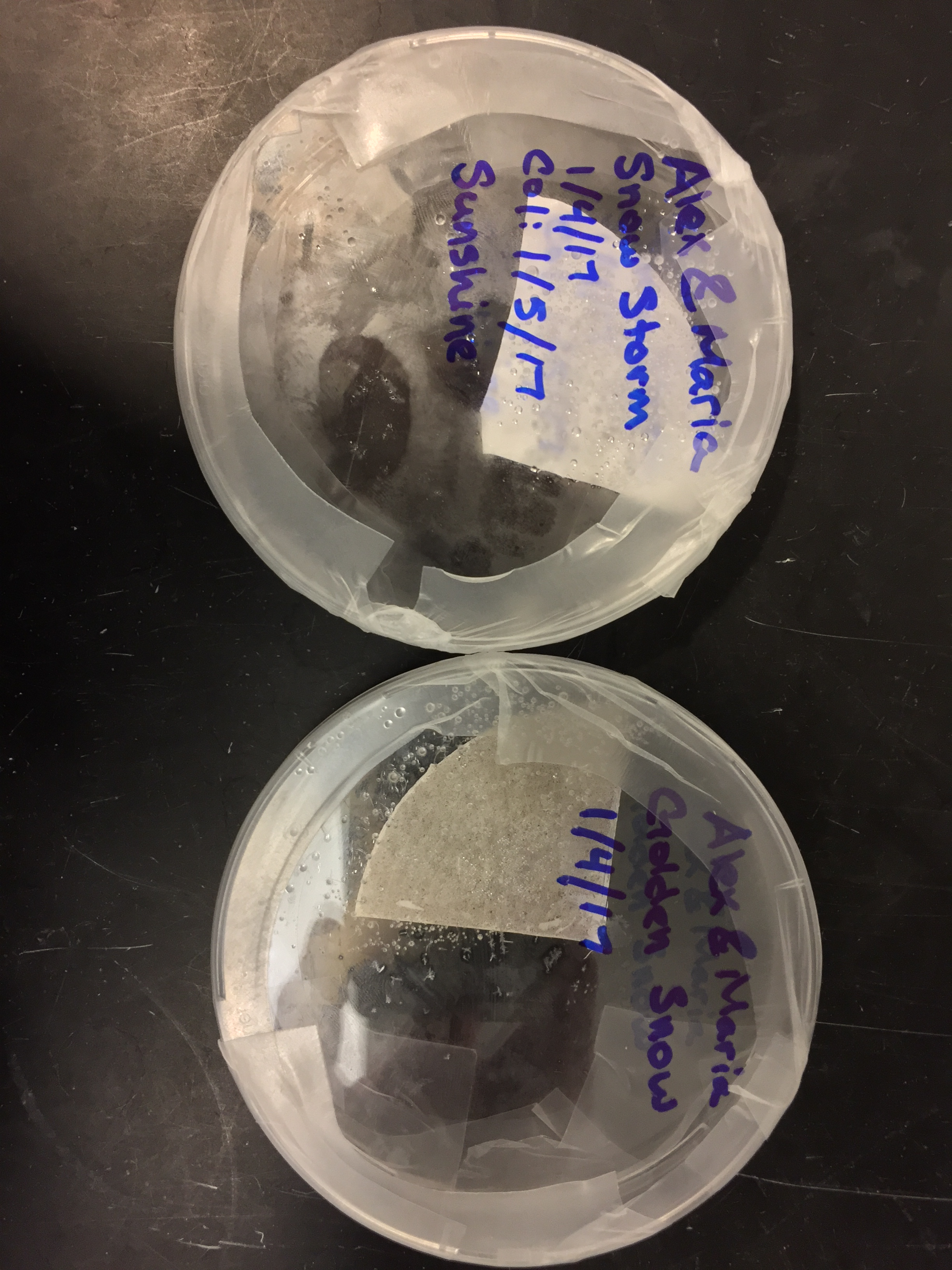

Supplement: Supplemental Information 7 — These two filter quadrants (0.45-μm pore size) collected snow from the same storm on 01/04/17. The top (cleaner) filter filtered snow from Sunshine; the bottom (dirtier) filter filtered snow from Golden. [file peerj-06-5961-s007.jpg]
